# Accidental Ingestion of a Novel Psychoactive Substance: A Case Report

**DOI:** 10.7759/cureus.11185

**Published:** 2020-10-26

**Authors:** Abhay Kant, Rupeng Mong, Hock Heng Tan

**Affiliations:** 1 Emergency Medicine, Changi General Hospital/Singhealth, Singapore, SGP; 2 Accident and Emergency, Changi General Hospital/Singhealth, Singapore, SGP

**Keywords:** novel psychoactive substances, 5-fluoro-mdmb-pica, synthetic cannabinoids, seizure, singapore

## Abstract

Novel psychoactive substances (NPSs) are a new generation of designer drugs that are quickly replacing the traditionally abused street drugs. Since their development, the number of molecules in NPSs and their variants have expanded exponentially. Little is known locally about the toxic effects of the exposure of these NPSs. We report two cases of accidental ingestion of methyl (2S)-2-{[1-(5-fluoropentyl)-1H-indole-3-arbonyl]amino}-3,3-dimethylbutanoate (5-fluoro-MDMB-PICA), a recent NPS. They were drinking the liquid in a winter melon tea bottle, and one patient had a seizure episode directly after ingestion. Both patients were managed supportively and discharged after a brief hospitalization period. Presentation to the emergency departments (EDs) following exposure to NPSs may become more common. Knowledge about the impact of NPS exposure and their clinical effects is lacking amongst emergency physicians in Singapore, and this case report serves as a potential resource for physicians.

## Introduction

The use and availability of novel psychoactive substances (NPSs) have grown rapidly over the last few years globally [1], and a similar pattern is being seen regionally. This has become a serious issue with an increasing number of cases of adverse effects and toxicities. There are multiple factors behind the trends in NPS distribution and usage. NPSs produce similar or stronger psychoactive effects compared to traditional street drugs of abuse. Moreover, newer analogs keep emerging from multiple geographical locations worldwide to keep up with demands and escape detection by law enforcement. While there are hundreds of NPSs currently in production, the most popular are the synthetic cannabinoids (SCs) and cathinones [1]. NPSs are sold as "herbal medications,” "bath salts,” "insect repellents,” "research chemicals," [2], "heart shot black," [3], or "air fresheners," with disclaimers like "not for human consumption" or "for research purpose only" to avoid legal hassles. In Singapore, in 2019, NPSs displaced cannabis as the second most commonly abused drugs among new abusers and became the third most commonly abused drug in any category. Methamphetamine, heroin, and NPS were the top three drugs for all abusers [4]. Therefore, NPSs represent much more than a healthcare burden alone.

We present two cases of inadvertent ingestion of methyl (2S)-2-{[1-(5-fluoropentyl)-1H-indole-3-arbonyl]amino}-3,3-dimethylbutanoate (5-fluoro-MDMB-PICA), a recent NPS.

## Case presentation

Case 1

A 67-year-old woman presented to the emergency department (ED) after the inadvertent ingestion of 5-fluoro-MDMB-PICA. She had a history of hypertension and hyperlipidemia and was on regular enalapril (20 mg once morning dose) and statins. She and her friend (case 2) were drinking from a winter melon tea bottle which contained a clear yellow, pungent fluid approximately 1.5 to two hours before presentation. She had upward rolling of her eyes with limb jerking lasting for a brief duration that ceased without intervention, approximately 45 minutes after the estimated ingestion time. The patient was healthy before the ingestion and had no history of seizure. When the paramedic team arrived at the scene, the patient was drowsy with a Glasgow Coma Scale (GCS) score of six (eye [E] 4, verbal [V] 1, motor [M] 1), and her capillary blood glucose was 7.3 mmol/L.

On examination upon arrival in the ED, she was afebrile (97.3 °F), tachycardic (heart rate [HR], 111 beats per minute [bpm]), and had a respiratory rate of 21 breaths per minute. Her blood pressure (BP) was 125/52 mmHg, and she had 95% oxygen saturation (SpO_2_) on 4 L/minute oxygen via nasal cannula. Her capillary blood glucose level was 6.7 mmol/L. Her GCS on arrival was three, and her pupils were 3 mm, reactive bilaterally. Her heart sounds were dual, and lungs sounds were clear with shallow breathing efforts. Her electrocardiogram (ECG) showed normal sinus rhythm. A venous blood gas analysis showed respiratory acidosis with a pH of 7.239, partial pressure of carbon dioxide (pCO_2_) of 68.4 mm Hg, and bicarbonate (HCO_3_) of 23.9 mmol/L. The renal panel, full blood count, liver function tests, serum amylase, serum lactate, serum osmolality, and coagulation profile were within reference range while serum ethanol, serum paracetamol, and salicylate levels were undetectable. The chest X-ray showed left retrocardiac consolidation with left pleural effusion suggestive of pneumonia. She was hypotensive (BP, 82/46 mmHg) for approximately one hour after her arrival. Fluid challenge via a crystalloid bolus of 1.5 L was administered, and she remained hypotensive (BP, 79/38 mmHg; HR, 100 bpm). Her GCS improved to 13 (E3V4M6) approximately 90 minutes after arrival. She appeared drowsy but was arousable and was able to follow simple commands.

**Figure 1:**
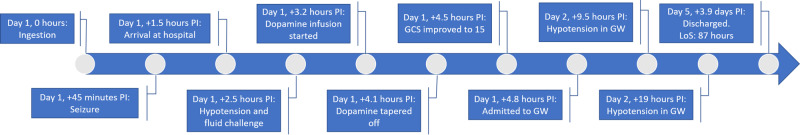
Case 1 timeline of events PI: post-ingestion, GCS: Glasgow Coma scale, GW: general ward, LoS: length of stay.

Her BP stabilized within the reference range following a dopamine infusion. She underwent a plain computed tomography scan of her brain, the findings of which were unremarkable. She was weaned off the dopamine infusion after approximately 45 to 60 minutes, and her GCS improved to 15 (approximately 4.5 hours after ingestion of an NPS). She was admitted to a general ward bed. She was discharged after an uneventful inpatient ward stay of approximately 87 hours. There were few episodes of hypotension (i.e., her systolic BP was in the 80 to 89 mmHg range) during the inpatient ward stay; these episodes were corrected with fluid boluses and required no pressor or inotropic support. She was treated for aspiration pneumonia with intravenous antibiotics. Her serial measurements of inflammatory markers (e.g., C-reactive protein and procalcitonin) remained low and insignificant. There were no episodes of hypoxia documented. The progression of events is summarized in Figure 1. 

Case 2

The second patient was a 79-year-old woman who was otherwise healthy with no relevant medical history. This patient drank from the same winter melon tea bottle that case 1 had used. While the exact amount of 5-fluoro-MDMB-PICA ingested was unknown, the inadvertent ingestion occurred approximately two hours before arriving at the hospital. She was noted to be unwell by her husband, rested on her bed, then vomited once and became unconscious. On arrival of the paramedics at the scene, she appeared drowsy with GCS of seven (E2V1M4) with greenish vomitus on her clothes, and her capillary blood glucose 7.6 mmol/L.

**Figure 2:**
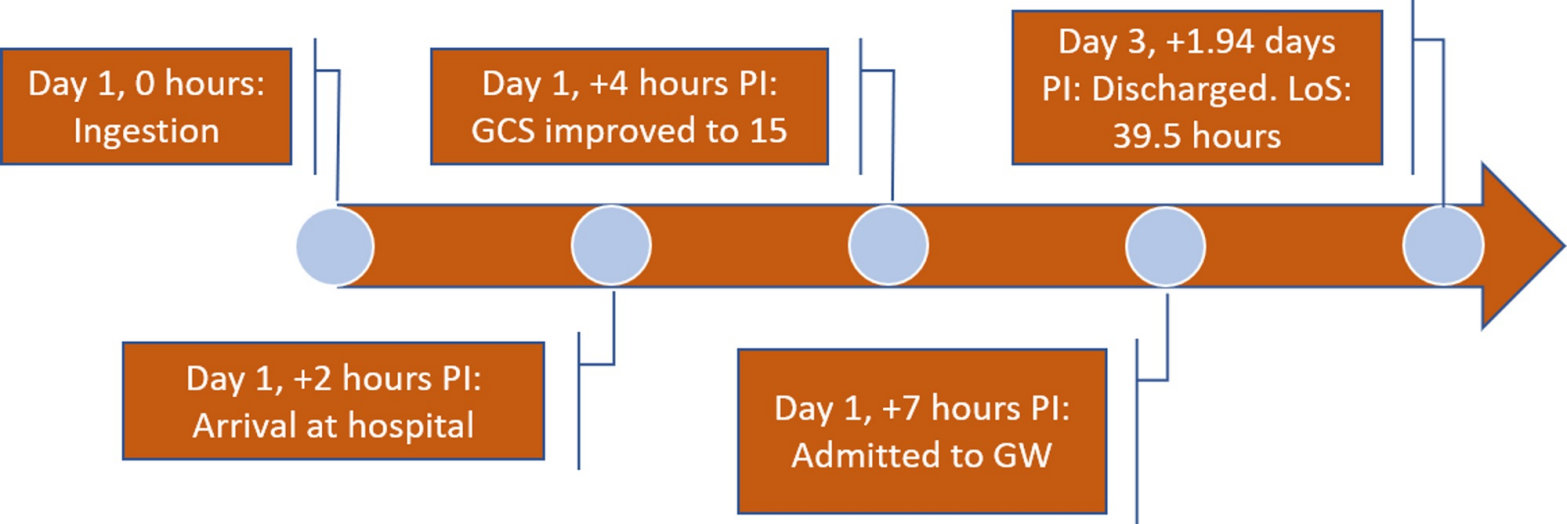
Case 2 timeline of events PI: post-ingestion, GCS: Glasgow Coma scale, GW: general ward, LoS: length of stay.

On examination in the ED, she was afebrile (98.6 °F), HR was 67 bpm, respiratory rate was 20 breaths per minute, PB was 113/71 mmHg, her SpO_2_ on room air was 100%, and her blood glucose level was 7.7 mmol/L. There was no incontinence, and she was not diaphoretic. Her initial GCS was eight (E2V1M5), and her pupils were 4 mm bilaterally and sluggish. There was no facial asymmetry, and she was moving all four of her limbs minimally. Her heart sounds were dual, and her lungs sounds were clear with poor breathing efforts. Her abdomen was soft. The prehospital paramedic team brought a bottle containing a liquid. Her ECG showed normal sinus rhythm with no tachycardia. Her venous blood gas analysis results were within reference ranges with a pH of 7.382, pCO_2_ of 46.7 mm Hg, and HCO_3_ of 25.7 mmol/L. The results of her renal panel, full blood count, liver function tests, serum amylase, serum osmolality, and coagulation profile were within reference ranges. Her serum ethanol, serum paracetamol, and salicylate levels were undetectable. Her chest X-ray showed some faint opacity in the left middle to lower zones. After approximately 2.5 hours in the ED, her GCS improved to 15, approximately four hours after the estimated time of ingestion. Her vital signs remained stable, and her respiratory efforts improved. She was admitted to the general ward. She was discharged after an uneventful inpatient ward stay of approximately 39.5 hours. She was treated for aspiration pneumonitis as well. The progression of events is summarized in Figure 2. 

Both patients' blood and urine specimens were sent for toxicology screens approximately 3.5 hours after their arrival. The laboratory processed the blood and urine specimens the next day, approximately 14 hours after being collected. The hydrolysis metabolite for 5-Fluoro-MDMB-PICA-Ester was detected in the urine in both patients (Table 1). No other drug of abuse or other drugs that could account for their presentation was detected in the detailed toxicology tests. The yellow liquid contained inside the plastic bottle from which these two patients were drinking was tested and contained N-(adamantan-1-yl)-1-(5-chloropentyl)-1H-indazole-3-carboxamide (5-chloro-APINACA), 5-Fluoro-MDMB-PICA, and acetone.

**Table 1:** Summary of both patient cases F: female, GCS: Glasgow Coma scale, ED: emergency department, 5-Fluoro-MDMB-PICA: methyl (2S)-2-{[1-(5-fluoropentyl)-1H-indole-3-arbonyl]amino}-3,3-dimethylbutanoate.

Case	Age (years)/Sex	Symptoms and Clinical Presentation	Management	Disposition From ED	Length of Hospital Stay and Outcome	Significant Blood Toxicology Findings	Significant Urine Toxicology Findings
1	67/F	Seizures, drowsiness with low GCS, hypotension	Fluid boluses, transient use of dopamine, antibiotics for aspiration pneumonia	Internal medicine general ward	87 hours, Discharged home	Nil	5-Fluoro-MDMB-PICA-ester hydrolysis metabolite, acetone
2	79/F	Syncope, vomiting, drowsiness with low GCS	Symptomatic, antibiotics for aspiration pneumonitis	Internal medicine general ward	27 hours, Discharged home	Nil	5-Fluoro-MDMB-PICA-ester hydrolysis metabolite

## Discussion

The United Nations Office on Drugs and Crime categorizes NPSs in nine classes consisting of phenethylamines, SCs, synthetic cathinones, piperazines, tryptamines, aminoindanes, phencyclidine-type substances, plant-based substances, and other substances [2,5]. 5-Fluoro-MDMB-PICA is in the SC class. The pharmacological effects and mechanisms of the NPSs are summarized as interacting with the opioid receptor and inhibitory neurotransmitters; activation of the cannabinoid receptor type 1 (CB1); the action of the neurotransmitter gamma-aminobutyric acid (GABA) at the GABA A receptor to produce sedative, hypnotic, and anxiolytic effects; modulation of the levels and actions of the monoamine neurotransmitters dopamine, epinephrine, and serotonin; action as an N-methyl-D-aspartate receptor antagonist; and action that mediates specific serotonin receptor activities [2]. Some substances have more than one mechanism of action. Essentially, their effects can be assigned under six main groups as stimulants (36%), SC receptor agonists (SCRAs; 30%), classic hallucinogens (15%), opioids (8%), sedatives and hypnotics (4%), and dissociatives (3%). Four percent of NPSs could not be assigned to any of these six main effect groups [2].

The NPS 5-fluoro-MDMB-PICA functions as an SCRA. SCs are human-made substances that bind to cannabinoid receptors in the human body. Compared to tetrahydrocannabinol (THC), which is the psychoactive alkaloid of cannabis and a partial agonist of cannabinoid receptors CB1 and CB2, SCs are full agonists of the receptors. SCs are sold as "Spice Gold,” "Spice Silver,” "Spice Diamond,” "K2,” "Bliss,” "Black Mamba,” "Bombay Blue,” "Blaze,” "Genie,” "Zohai,” "JWH-018 (-073, (-250),” "Kronic,” "Yucatan Fire,” "Skunk,” "Moon Rocks," and "Mr. Smiley" [6]. SCs are a big group of chemically diverse agents and have structures unrelated to D9-THC, different metabolism, and greater toxicity. Despite marked differences in their chemical structure, they are usually lipid-soluble, nonpolar, and typically consist of 20 to 26 carbon atoms, which somewhat explains their volatile nature and ease in which they can be smoked. Fluorine substitution at the 5-pentyl position of pentylindole/pentylindazole, a recently popular structural modification of SCs, generally enhances compounds' potency and stability and prolongs half-life [7]. However, the complete details regarding their pharmacokinetics and metabolism are not available, barring selected laboratory studies. Likely, the original 5-chloro-APINACA, 5-fluoro-MDMB-PICA compounds present in the plastic bottle were metabolized after ingestion by the patients, and only the 5-fluoro-MDMB-PICA-ester hydrolysis metabolite could be detected in the urine sample of both patients. Interestingly, the two fatal cases associated with consumption of 5-fluoro-MDMB-PICA in the report of Chan et al. [8] also found only ester hydrolysis metabolites. This NPS is likely present in low concentrations and is rapidly metabolized.

Adverse effects may be cardiovascular (e.g., sympathomimetic effects such as tachycardia, palpitations, hypertension, myocardial infarction, and arrhythmias), neurological (e.g., seizures, tremors, stroke, fear, aggressive behavior, changing moods, and confusion [9]), gastrointestinal (e.g., nausea and vomiting), psychological (e.g., agitation, confusion, drowsiness, lethargy, hallucination, irritability, and paranoia), pulmonary (e.g., respiratory depression), metabolic, or muscular [10,11]. However, unlike cannabis, SCs have significant potential to cause severe and life-threatening toxicity. These substances are available as a powder to smoke and pills and liquids for oral ingestion. The manufacturing process is unregulated, and the substances often contain impurities consisting of chemicals in different concentrations, making it very difficult to determine substance-specific effects. The agent in our cases, 5-fluoro-MDMB-PICA, has been encountered in powdered form and as a synthetic constituent in herbal plant mixtures most commonly distributed for smoking or vaping [12]. Also, 5-fluoro-MDMB-PICA is not known to have any therapeutic uses.

Seizures have been reported after NPS use, but the etiology is not fully clear. Mortality after their usage has been reported locally by Chan et al. [8] in 2019 where this substance was found in the postmortem blood toxicology analysis. All the NPS-related fatalities encountered in Singapore from 2016 to 2018 were due to either 2-(4-Bromo-2,5-dimethoxyphenyl)-N-[(2-methoxyphenyl) methyl]ethanamine (abbreviated as 25B-NBOMe) or SCs such as N-(1-amino-3,3-dimethyl-1-oxobutan-2-yl)-1-(4-fluorobenzyl)-1H-indazole-3-carboxamide (abbreviated as ADB-FUBINACA) and methyl 2-[1-(5-fluoropentyl)-1H-indazole-3- carboxamide]-3,3-dimethylbutanoate (abbreviated as 5-fluoro ADB; also known as 5-fluoro-MDMB-PINACA). According to Chan et al., 5-fluoro-ADB may be more toxic to humans than other SCs [8]. Seizures caused by SCs warrant initial treatment with benzodiazepines (e.g., lorazepam) with repeat doses as necessary [13]. Other severe intoxication caused by SCs can be life-threatening and warrants prompt treatment directed at the most significant clinical diagnosis and findings [13]. Treatment is mostly supportive, and no specific antidote therapy is indicated or available as of this writing. There are diagnostic challenges to a physician when encountered with a patient presenting with signs and symptoms of toxicity with NPSs or SCRAs in the absence of a definitive history of ingestion, as the presentation is often variable in different patients with no definite toxidrome. Definitive diagnosis is currently only via blood and urine toxicology testing, which have a longer turnaround time and are not available readily in most local hospital laboratories. This makes immediate clinical management decisions based on the laboratory toxicology testing ineffective.

Although most cases of poisonings and toxicity have co-ingestions, these two cases most likely represented isolated ingestion of the NPS, based on their self-reported histories and the drug and urine reports obtained later. Our two cases concur with the common adverse effects of NPSs. We could infer from the timeline and clinical presentation of our patients that 5-fluoro-MDMB-PICA can cause altered mental state and emesis and, in more severe poisoning, may lead to seizure and cardiovascular instability. However, these effects are likely to be self-limiting, and patients would likely respond to supportive medical therapy.

The use of NPSs is continuing to evolve and expand globally. Locally, NPSs are among the three most abused drugs [4]. There are substantial challenges that the identification and control of such substances pose to effective health and law enforcement regulation. Labels on packages and actual constituents of the product are often inaccurate or mismatched [5]. Recently, these substances have been introduced in the market through various modes of distribution, including the Internet, headshops or "smart shops" that sell drug paraphernalia, or street-level drug traffickers as legal alternatives to illicit drugs, accounting for an increasingly significant share of illicit drug markets, all of which become a matter of great concern to public health [6].

## Conclusions

The use of NPSs is on the rise, and they may become the most commonly abused street drugs, both locally and globally. Despite their shared category as NPSs for clinical discussion parlance, the diversity in these substances is unlike any other group of drugs. In the context of NPSs, essential differences between classes of substances are noted. Emergency physicians will need to be aware of their toxicity, presentation, and the type of management involved in treating these patients. Understanding and keeping abreast of the developments of this big class of drugs may present a challenge, but these case descriptions should provide illustrative examples for other physicians.

## References

[1] (2020). UNODC early warning advisory on new psychoactive substances. https://www.unodc.org/LSS/Page/NPS.

[2] (2020). UNODC new psychoactive substances portal and international collaborative exercise portal. https://www.unodc.org/LSS/Home/BothAreas.

[3] Usui K, Fujita Y, Kamijo Y, Kokaji T, Funayama M (2018). Identification of 5-fluoro ADB in human whole blood in four death cases. J Anal Toxicol.

[4] (2020). Central Narcotics Bureau news release. https://www.cnb.gov.sg/docs/default-source/drug-situation-report-documents/cnb-annual-statistics-2019.pdf.

[5] Rhumorbarbe D, Morelato M, Staehli L, Roux C, Jaquet-Chiffelle DO, Rossy Q, Esseiva P (2019). Monitoring new psychoactive substances: exploring the contribution of an online discussion forum. Int J Drug Policy.

[6] (2020). United Nations Office on Drugs and Crime: the challenge of new psychoactive substances. https://www.unodc.org/documents/scientific/NPS_Report.pdf.

[7] Zawilska JB, Andrzejczak D (2015). Next generation of novel psychoactive substances on the horizon: a complex problem to face. Drug Alcohol Depend.

[8] Chan S, Wu J, Lee B (2019). Fatalities related to new psychoactive substances in Singapore: a case series. Forensic Sci Int.

[9] Kleis J, Germerott T, Halter S, Heroux V, Roehrich J, Schwarz CS, Hess C (2020). The synthetic cannabinoid 5F-MDMB-PICA: a case series. Forensic Sci Int.

[10] Bae K, Kwon NJ, Han E (2018). A review on the abuse of three NPS (synthetic cannabinoids, kratom, poppers) among youths in Asia. Forensic Sci Int.

[11] Gatch MB, Forster MJ (2019). Cannabinoid-like effects of five novel carboxamide synthetic cannabinoids. Neurotoxicology.

[12] (2020). World Health Organization: critical review report 5F-MDMB-PICA. https://www.who.int/medicines/access/controlled-substances/Final_5F-MDMB-PICA.PDF?ua=1.

[13] (2020). Synthetic cannabinoids: acute intoxication. https://www.uptodate.com/contents/synthetic-cannabinoids-acute-intoxication.

